# Are There Similarities in Pregnancy Complications and Delivery Outcomes among Sisters?

**DOI:** 10.3390/jcm11226713

**Published:** 2022-11-13

**Authors:** Hila Shalev-Ram, Gal Cohen, Shai Ram, Lior Heresco, Hanoch Schreiber, Tal Biron-Shental, Michal Kovo, Dorit Ravid

**Affiliations:** 1 Department of Obstetrics and Gynecology, Meir Medical Center, Kfar Saba 4428164, Israel; 2 Department of Obstetrics and Gynecology, Sackler Faculty of Medicine, Tel Aviv University, Tel Aviv 6195001, Israel; 3 Department of Obstetrics and Gynecology, Lis Maternity Hospital, Sourasky Medical Center, Tel Aviv 6423906, Israel

**Keywords:** sisters, familial predisposition, operative delivery, dysfunctional labor, nullipara

## Abstract

This retrospective cohort study evaluated pregnancy outcomes and similarities between pairs of nulliparous sisters with a singleton fetus who delivered between 2013 and 2020. The “Sister-1 group” was defined as the sibling who delivered first, while the “Sister-2 group” included the siblings who gave birth after Sister-1. Obstetrical complications and delivery outcomes were compared. The relative risk for recurrence of a complication in Sister-2 was calculated. The study included 743 sister pairs. There were no between-group differences in maternal BMI, gestational age at delivery, gravidity, smoking, or epidural rates. The Sister-2 group was older than the Sister-1 group (26.4 ± 5 vs. 25.8 ± 4.7 years, respectively, *p* = 0.05). Higher birthweights and more large-for-gestational-age infants characterized the Sister-2 group compared with the Sister-1 group (3241 ± 485 g vs. 3148 ± 536 g, *p* < 0.001 and 7.7% vs. 4.8%, *p* = 0.025, respectively). There were no between-group differences in the rate of small-for-gestational-age, gestational diabetes, hypertensive disorders, pre-term births, vacuum extraction, or cesarean deliveries. Logistic regression analysis found that if Sister-1 underwent vacuum extraction, her sibling had an increased risk for vacuum delivery (adjusted RR 3.03, 95% CI 1.4–6.7; *p* = 0.003) compared with those whose sibling (Sister-1) did not. There was a three-fold risk of vacuum extraction delivery between sisters. This finding could be related to biological inheritance, environmental factors, and/or psychological issues that may affect similarities between siblings’ delivery outcomes.

## 1. Introduction

Complex interactions between genetic and environmental factors may affect pregnancy complications and labor patterns. Several studies have investigated familial pregnancy patterns, specifically, similarities between pregnancy complications that mothers and daughters might share. An excess occurrence in mothers and their daughters has been observed in pre-eclampsia [1], prolonged pregnancy [2,3], pre-term birth [4], labor dystocia [5,6], and vaginal-birth failure [7], as well as correlations between birthweight [8] and long duration of labor [9].

Significant differences in the management of pregnancy and childbirth between women of different generations may affect the results in mothers and daughters. Like mothers and daughters, sisters also share a 50% similarity in their genetic load. They also tend to share similar socio-demographic backgrounds and environmental influences.

Data regarding similarities and differences between sisters in labor patterns, as well as in the mode of delivery, are sparse. A study conducted by Tollanes et al. found that a younger sister whose older sister had her first child by cesarean delivery (CD) had a higher chance of having a CD as well [7]. It was also found that the rates of labor dystocia are similar among sisters [5,6]. Others observed a correlation between pre-pregnancy BMI and weight gain during pregnancy among sisters and a weak correlation in the birth weights of babies born to sisters [10]. It was also found that the risk for pre-term birth was higher if the first sister had a pre-term delivery [11].

The current study aimed to broaden the knowledge regarding pregnancy complications and birth patterns among sisters. We explored this correlation using linked birth data between sisters recorded in the birth registry of a large tertiary medical center in Israel. This enabled us to compare genetically related women who were close in age and who received similar care under similar conditions and medical guidelines during their pregnancies and deliveries. 

## 2. Materials and Methods 

This population-based retrospective cohort study used data retrieved from the electronic healthcare records of patients who delivered at Meir Medical Center, Kfar Saba, Israel between 2013 and 2020. 

Data were collected from the electronic medical records and included information on maternal demographics and medical, obstetric, and prenatal history, as well as information about pregnancy complications, labor, and delivery. Data regarding early neonatal outcomes were also collected.

We identified sisters by pairing women with the same maiden name, parental names, and maternal and paternal origins. In the case of pairs of sisters with the same paternal names and same maternal and paternal origins but different family names, we contacted them directly by telephone and asked if they were indeed sisters. If more than two sisters were identified, we paired the sister who gave birth first with the other sister (Sister-1 was compared with Sister-2 and Sister-1 was compared with Sister-3). For the purpose of the study, the sister who had her first pregnancy earlier, regardless of age, was defined as “Sister-1”, while the sister who gave birth second was defined as “Sister-2”.

### 2.1. Ethics

The Institutional Review Board approved the study protocol and the Ethics Committee waived the requirement for informed consent as the data were fully anonymized before analysis. Included were nulliparous sisters, 18 to 40 years old, who delivered at Meir Medical Center and who had well-documented data regarding their pregnancies and deliveries. An additional inclusion criterium was the birth of a singleton viable fetus (beyond 24 weeks of gestation) without fetal anomalies or genetic abnormalities.

### 2.2. Outcomes

The data were used to determine whether sisters of women with a complicated pregnancy had a higher risk of developing the same complication, compared with sisters of women with uncomplicated pregnancies. The outcomes of interest were pregnancy and obstetrical complications. We compared the relative risk for gestational diabetes mellitus (GDM) A1 and A2; hypertensive disorders (pre-eclampsia [12] and chronic hypertension); pre-term birth (PTB, defined as <37 and <34 weeks of gestation); small-for-gestational-age infants (SGA, defined as neonatal birthweight < 10th percentile based on Israeli growth charts) [13]; large-for-gestational-age infants (LGA, defined as birth weight >90th percentile) [13] in the sister of a woman with this condition compared with a sister of a woman without it. We also compared the risk of vacuum extraction (VE) and CD.

### 2.3. Statistical Analysis

The two groups of sisters were compared using a chi-square test for categorical variables and independent *t*-tests for the continuous variables, to ensure that they had similar demographic characteristics. Next, we identified the incidence of pregnancy complications in the two groups. Last, we compared the relative risk for the sibling (Sister-2 group) of women with pregnancy and/or delivery complication (Sister-1 group) to have the same complication compared with the siblings of women without this condition. *p* < 0.05 was considered statistically significant. All analyses were performed using SPSS, version 23 (IBM Corp., Armonk, NY, USA). Logistic and multinomial logistic regression analyses were used to estimate relative risks and 95% confidence intervals. Probability values were determined by nonparametric tests of the medians.

## 3. Results

During the study period, 4503 pairs of sisters were found; among them were 777 (17.3%) pairs of nulliparous women, of which 743 pairs (16.5%) met the inclusion criteria. Baseline characteristics of the sisters were divided into those who had given birth first (Sister-1 group) and those who had given birth second (Sister-2 group) are shown in Table 1. There were no significant differences in BMI between the groups before and after pregnancy, gestational age at delivery, gravidity, smoking, and epidural rates. Sisters who gave birth second were older than sisters who gave birth first (26.4 ± 5 vs. 25.8 ± 4.7 years, respectively; *p* = 0.05).

Table 2 outlines maternal and neonatal characteristics of the study groups. Birth weights and LGA rates were higher among the Sister-2 group compared with the Sister-1 group (3241 ± 485 g vs. 3148 ± 536 g, respectively, *p* < 0.001, and 57 (7.7%) vs. 36 (4.8%), respectively, *p* = 0.025). SGA rates did not differ between the groups. GDM, hypertensive disorders, prematurity rates, VE rates, and urgent CD rates were found to be similar between groups.

The relative risk analysis is shown in Table 3. A woman whose sister had a VE had a three-fold risk of a vacuum delivery (adjusted RR 3, 95% CI: 1.4–6.7). Relative rates of other complications including pre-term births, GDM, hypertensive disorders, LGA and SGA, CDs, and urgent CDs were similar between the groups.

## 4. Discussion

The current study identified a significant association of instrumental deliveries between sisters, with a three-fold risk for VE in women whose sisters underwent vacuum delivery. 

We performed a comprehensive comparison of pregnancy complications and delivery outcomes among sisters in their first delivery. Regarding pregnancy complications, data concerning associations of GDM and pre-eclampsia between sisters are lacking. It is well-established that women with a family history of diabetes have a higher risk for GDM [14], but whether there is an association of GDM between sisters is not known. In the current study, we did not find an increased risk for GDM among women with sisters who had GDM or an increased risk for pre-eclampsia among sisters. Notably, Skjærven et al. showed that sisters of women who were themselves born after pregnancies of their mothers that were complicated by pre-eclampsia had about twice the risk of pre-eclampsia in their pregnancies [1], implying heritable aspects of pre-eclampsia that are not yet fully understood. They did not investigate whether sisters of a woman who had pre-eclampsia during her own pregnancy had an elevated risk for pre-eclampsia. 

Regarding labor outcomes, studies among relatives demonstrated a higher incidence of labor dystocia among mothers and daughters, as well as between sisters [5,6]. We demonstrated an increased risk (adjusted RR = 3) for vacuum delivery among sisters. Our findings are in accordance with a study by Bers-Lakas et al. [6], who reported more than triple the risk (OR = 3.5) for an assisted instrumental delivery among women having older sisters who underwent instrumental intervention and more than a 20-fold risk (OR = 24) among twins [6]. However, their study included women who delivered between 1955 and 1972 in various medical centers, so the relevance to contemporary practice is questionable. 

Several mechanisms may contribute to this type of familial association, including genetic influences, environmental factors, and/or psychological parameters. Using model fitting, Algovik et al. suggested that genetic effects account for 28% of susceptibility for labor dystocia [5]. Non-genetic familial factors may also contribute to similarities between sisters. For example, unfavorable experiences of close relatives may influence anxiety levels during pregnancy and delivery, which in turn may influence labor progression and mode of delivery [15,16]. 

In the current study, we did not observe an association between CD or pre-term birth between sisters; yet, other studies showed familial associations in CD rates [6,7]. According to Tollånes et al. [7], a younger sister whose older sister had her first child delivered by CD had a 45% increased risk of having her first child by CD (RR 1.45, 95% CI 1.40–1.51). 

The current study had several strengths. First, all deliveries were managed at the same university medical center during a period of 7 years, which prevents bias arising from changing guidelines and different clinical approaches across medical centers. Additionally, data and familial relationships were validated through telephone questionnaires.

The main limitation of the current study lies in its retrospective nature. A larger cohort may have enabled us to demonstrate additional statistically significant differences. Another weakness is the lack of data on some possible confounders such as maternal height or clinical pelvimetry. Nonetheless, our cohorts were similar in the main characteristics known to affect the risk for instrumental delivery, such as maternal age, BMI, and weight gain during pregnancy. 

In conclusion, of all the complications that were investigated between sisters, we found an increased risk for vacuum delivery among sisters. Importantly, while our data showed an increased risk of vacuum delivery among sisters, the strength and assuredness of this association is not completely clear and needs further evaluation. This type of association should be added to the various considerations related to patient counseling and delivery management. Further research is needed to explore the possible genetic and psychological influences underlying associations between pregnancy and delivery outcomes among sisters. 

## Figures and Tables

**Table 1:** Baseline characteristics of sisters.

Characteristic	Sister-1 Group *n* = 743	Sister-2 Group *n* = 743	*p*-Value
Maternal age at delivery, years (mean ± SD)	25.8 ± 4.7	26.4 ± 5	0.05
BMI before pregnancy, kg/m^2^ (mean ± SD)	23.7 ± 5.2	23.6 ± 5.1	0.76
BMI after pregnancy, kg/m^2^ (mean ± SD)	28.5 ± 5.7	28.4 ± 6.2	0.8
Gestational age at delivery, weeks, (mean ± SD)	38.8 ± 1.8	39 ±1.6	0.08
Gravidity (mean ± SD)	1.2 ±0.5	1.2 ±0.5	0.22
Smoking, *n* (%)	28 (3.8%)	26 (3.5%)	0.78

BMI—body mass index; SD—standard deviation.

**Table 2:** Sisters’ obstetrical and neonatal characteristics.

Characteristic	Sister-1 Group *n* = 743	Sister-2 Group *n* = 743	*p*-Value
Epidural, *n* (%)	465 (62.6%)	433 (58.3%)	0.09
Gestational diabetes mellitus (A1 + A2), *n* (%)	63 (8.5%)	84 (11.3%)	0.07
Hypertensive disorders	30 (4%)	26 (3.5%)	0.58
Neonatal birthweight, g (mean ± SD)	3148 ± 536	3241 ± 485	<0.001
Large for gestational age, *n* (%)	36 (4.8%)	57 (7.7%)	0.025
Small for gestational age, *n* (%)	45 (6.1%)	31 (4.2%)	0.1
Pre-term birth <34 w, *n* (%)	14 (1.9%)	9 (1.2%)	0.29
Pre-term birth <34–37 w, *n* (%)	79 (10.6%)	71 (9.6%)	0.49
Vacuum extraction *n* (%)	58 (7.8%)	48 (6.5%)	0.31
Cesarean delivery *n* (%)	163 (21.9%)	140 (18.9%)	0.142
Urgent Cesarean delivery, *n* (%)	99 (13.3%)	75 (10.1%)	0.054

**Table 3:** Relative risk of pregnancy and delivery outcomes across sisters.

Variable	Prevalence of Complications in Sister-2 Group		Crude Relative Risk (95% CI)	Adjusted Relative Risk * (95% CI)
	Sister-1 Group without Complication	Sister-1 Group with Complication		
GDM, *n* (%)	76/680 (11.1%)	8/63 (12.6%)	1.1 (0.6–2.2)	1.0 (0.5–2.3)
Hypertensive disorders, *n* (%)	29/688 (4.2%)	1/25 (4%)	1.0 (0.1–6.8)	1.0 (0.1–7.2)
Large for gestational age, *n* (%)	33/653 (5.1%)	3/54 (5.5%)	1.1 (0.4–3.3)	1.1 (0.3–3.7)
Small for gestational age, *n* (%)	44/668 (6.6%)	1/30 (3.3%)	0.5 (0.07–3.8)	0.5 (0.07–3.8)
Pre-term birth <34 weeks, *n* (%)	8/729 (1.1%)	1/14 (7.1%)	6.6 (0.81–59.5)	8.3 (0.9–77.3)
Pre-term birth <34–37 weeks, *n* (%)	63/664 (9.5%)	8/79 (10.1%)	1.1 (0.5–2.31)	1.1 (0.5–2.23)
Pre-term birth <37, *n* (%)	75/635 (11.8%)	15/108 (13.8%)	1.2 (0.7–2.0)	1.2 (0.7–2.1)
Cesarean delivery, *n* (%)	107/580 (18.4%)	34/153 (20.9%)	1.1 (0.8–1.6)	1.2 (0.7–2.5)
Vacuum delivery, *n* (%)	39/658 (5.7%)	9/58 (15.5%)	2.7 (1.3–6.7)	3.0 (1.4–6.6)
Urgent CD, *n* (%)	63/644 (9.8%)	12/99 (12.1%)	1.2 (0.7–2.3)	1.3 (0.7–2.5)

GDM—gestational diabetes mellitus (A1 + A2); hypertensive disorders including chronic hypertension, gestational hypertension, and pre-eclampsia. * Adjusted for maternal age and neonatal birth weight.
